# In Vivo Confocal Microscopy Observation of Cell and Nerve Density in Different Corneal Regions with Monocular Pterygium

**DOI:** 10.1155/2020/6506134

**Published:** 2020-03-23

**Authors:** Yun-Zhi Shen, Mi Xu, Song Sun

**Affiliations:** Department of Ophthalmology, The Affiliated Wuxi No. 2 People's Hospital of Nanjing Medical University, Wuxi 214000, Jiangsu, China

## Abstract

**Purpose:**

To investigate the effects of pterygium on corneal cell and nerve density in patients with unilateral pterygium using in vivo laser scanning confocal microscopy (LSCM).

**Methods:**

In this cross-sectional study, 24 patients with unilateral pterygium who were treated in the Department of Ophthalmology of the Second People's Hospital of Wuxi City from April 2018 to July 2018 were analyzed. Each eye with pterygium and its fellow eye were imaged by LSCM. The density of basal corneal epithelial cells, anterior stromal cells, posterior stromal cells, dendritic cells, and endothelial cells in pterygium and adjacent clear cornea was measured. In the fellow eyes, the central cornea, nasal cornea, nasal mid-peripheral cornea, and temporal cornea were imaged. The difference in the density of cells and subepithelial nerve fibers in different corneal regions of eyes with pterygium was analyzed. The cell and nerve density of the fellow cornea were also measured to exclude the influencing factors.

**Results:**

The density of corneal basal epithelial cells in the central corneas of eyes with pterygium was 6497 ± 1776 cells/mm^2^, which was higher than that in the area near the head of pterygium (5580 ± 1294 cells/mm^2^, *P* < 0.001), the region above pterygium (6097 ± 1281 cells/mm^2^, *P*=0.049), and the region below pterygium (5463 ± 1007 cells/mm^2^, *P*=0.001). The density of anterior stromal cells in the central cornea was 742 ± 243 cells/mm^2^, which was higher than that in the area near the head of pterygium (587 ± 189 cells/mm^2^, *P*=0.005), the region below pterygium (492 ± 159 cells/mm^2^, *P*=0.005), and the temporal cornea (574 ± 164 cells/mm^2^, *P*=0.003). The density of endothelial cells in the central cornea was 2398 ± 260 cells/mm^2^, which was higher than that in the area near the head of pterygium (2296 ± 231 cells/mm^2^, *P*=0.011) and the region below pterygium (2272 ± 400 cells/mm^2^, *P*=0.020). The density of dendritic cells in the central cornea was 53 ± 48 cells/mm^2^, which was lower than that in the area near the head of pterygium (250 ± 224 cells/mm^2^, *P*=0.001), the upper region (103 ± 47 cells/mm^2^, *P*=0.006), and the lower region (90 ± 48 cells/mm^2^, *P*=0.023). The corneal nerve fiber length (CNFL) in the center was higher than that in the area near the head of pterygium, the upper region, and the lower region. Compared with fellow eyes, eyes with pterygium had a significantly higher mean corneal power (KM) (*P* < 0.001). There was a significant positive linear relationship between the corneal area invaded by pterygium of pterygia and KM (*r* = 0.609, *P*=0.009).

**Conclusion:**

Basal epithelial cells, stromal cells, endothelial cells, dendritic cells, and subepithelial nerve fibers in the central cornea of eyes with pterygium were different from those of pterygium and adjacent clear cornea. LSCM is effective for observing the morphology and quantity of corneal cells in pterygium.

## 1. Introduction

Pterygium is a common ocular surface disease that is characterized by the proliferation of bulbar conjunctiva and subconjunctival fibrovascular tissue in the palpebral fissure, which invades the clear cornea. Pterygium can cause corneal refractive disorders and visual impairment. The histopathological characteristics of pterygium are abnormal cell proliferation, inflammatory cell infiltration, fibrosis, angiogenesis, tissue degeneration, and excessive deposition of extracellular matrix. However, most of these pathological data come from studies of excised pterygium, and there are few reports on the pathological changes in pterygium in vivo, especially in adjacent corneal tissue. In this study, 24 eyes with pterygium were observed by in vivo confocal microscopy (IVCM) and compared with the fellow eyes. The reports are presented in the following.

## 2. Materials and Methods

### 2.1. Subjects

From April 2018 to July 2018, 24 eyes of 24 patients with a single pterygium examined in the Second People's Hospital of Wuxi City were recruited for this prospective study. All of the patients were ≥18 years of age. A comprehensive ophthalmic examination, including slit lamp examination, noncontact tonometry, and fundus examination, was performed for all participants. The exclusion criteria were bilateral pterygium; pseudopterygium; small pterygium that did not invade the corneal limbus; pterygium accompanied by chronic dacryocystitis, trachoma, blepharitis, and other ocular surface inflammation; pterygium accompanied by diabetes mellitus and other systemic diseases; ophthalmic surgery history; trauma history; fundus disease; contact lens use history; recent history of local special ophthalmic medication, glaucoma, or intraocular pressure >21 mmHg; central endothelial cell density (ECD) < 1800 cells/mm^2^ and mean corneal power (KM) on the healthy side >2D.

### 2.2. Anterior Segment Photography

The anterior segment of the affected eyes with pterygium and the fellow eyes was photographed with a slit lamp with a photo system.

### 2.3. Corneal Topography

The KM and corneal astigmatism (AST) of both the affected eyes and the fellow eyes in the 3.00 mm central zone were measured (Humphrey AILAS Corneal Topography System, Carl Zeiss Meditec, Inc., USA).

### 2.4. In Vivo Laser Scanning

**Figure 1:**
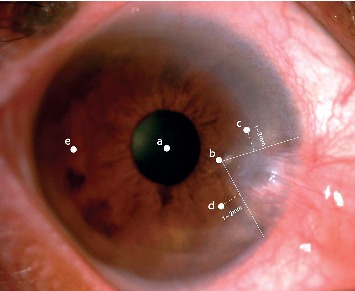
Measuring points: a: clear central cornea; b: clear cornea adjacent to the head of pterygium; c: upper cornea adjacent to the pterygium; d: lower cornea adjacent to the pterygium; e: temporal cornea.

Confocal microscopy (the Heidelberg Retina Tomograph/Rostock Cornea Module (HRT-II, Heidelberg Rostock Cornea Module, Heidelberg Engineering Inc, Germany)) was used in this study. Before the examination, 1 drop of topical anesthetic solution (oxybuprocaine hydrochloride eye drops, Santen Pharmaceutical Co., Ltd. Japan) was instilled onto the ocular surface. After proper preparation, one drop of carbomer eye gel (Dr. Gerhard Mann, Chempharm Fabrik GmbH, Germany) was applied to the surface of a sterile disposable plastic cap (Tomo-cap, Heidelberg Engineering GmbH) in the front lens of the microscope. Each eye with pterygium was measured in the following areas: clear central cornea; clear cornea adjacent to the head of pterygium; upper and lower cornea adjacent to the pterygium and temporal cornea (Figure 1); fellow cornea in central cornea, nasal cornea, nasal mid-peripheral cornea, and temporal cornea. The central area was excluded if pterygium invaded the central cornea. At least 3 confocal scans of the cornea were acquired at each examination.

### 2.5. Image Analysis

Rostock operation software was used to calculate the average density of corneal basal epithelial cells, anterior stromal cells (stromal layer close to Bowman's membrane), posterior stromal cells (stromal layer close to Descemet's membrane), and endothelial cells. Three images were analyzed at each observation point, and the average density was obtained. Three clear images (without motion blur or compression lines) from different observation points were selected for cell number counting. The cells were counted using Cell Count software (Heidelberg Engineering GmbH) in manual mode, and the average density was obtained. The data are expressed as density (cells/mm^2^) standard deviation.

The percentage and length of pterygium invading cornea were calculated by ImageJ software, a Java-based image processing software (National Institutes of Health, Bethesda, MD). The data were measured 3 times, and the mean results were used for analysis. Images of subepithelial nerve fibers with optimal quality from the IVCM examination with a depth of 40–80 *μ*m were chosen for analysis. The nerve fibers were manually labeled and automatically measured by ImageJ software with a plug-in NeuronJ (Biomedical Imaging Group, Lausanne, Switzerland). The following corneal nerve parameters were calculated: (1) corneal nerve fiber density (CNFD): the number of nerve trunks per square millimeter; (2) corneal nerve fiber length (CNFL): the length of the main nerves and branches per square millimeter; (3) corneal nerve branch density (CNBD): the number of nerve branches per square millimeter; (4) corneal nerve fiber tortuosity (CNFT): curvature of total nerve fibers, divided into grade 1∼4 according to the classification described by Olivera-Soto and Efron [1]. Three images were analyzed at each observation point, and the average result was obtained. All statistics were completed by the same operator.

## 3. Statistical Analysis

Statistical analysis was performed using SPSS 20.0 software. The Kolmogorov–Smirnov test was applied to all data samples in order to check the normal distribution. Measurement data are expressed as mean ± SD. Paired *t*-test was used to compare the corneal cell data and nerve parameters of each layer of the eyes with pterygium and the fellow eyes. A *P* value of less than 0.05 was considered statistically significant. The Pearson correlation test was performed to calculate the correlation between the percentage of pterygium to cornea and a decrease or increase in corneal ECD and between AST and a decrease in corneal ECD.

## 4. Results

From April 2018 to July 2018, 24 patients (24 eyes) met the criteria for this study, including 11 males (45.8%) and 13 females (54.2%). The age ranged from 39 to 83 years, with an average of 60 ± 12 years. All patients had pterygium located at the nasal side, with the head invading the cornea by 0.5 to 4 mm. The percentage of pterygium to cornea ranged from 1.9% to 26.6%, with an average of 12.05% ± 6.91%. The AST of eyes with pterygium in the 3.00 mm central zone ranged from 0.28 to 4.97D, with an average of 2.486 ± 1.266D, which was significantly higher than that of the fellow eyes. The KMs of the eyes with pterygium and the fellow eyes were not significantly different (Table 1).

**Figure 2:**
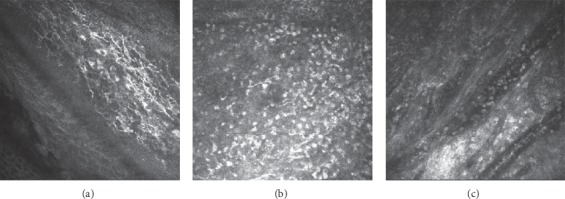
(a) Numerous dendritic cells in the pterygium head (depth = 24 *μ*m). (b) Numerous inflammatory cells in the pterygium head (depth = 33 *μ*m). (c) Fibrous and blood vessel proliferation (depth = 62 *μ*m).

**Figure 3:**
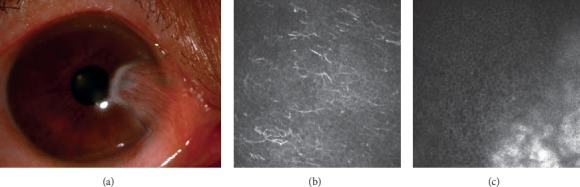
(a) Slit lamp photograph of corneal degeneration around the head of pterygium. (b) Activated dendritic cells infiltration (depth = 17 *μ*m). (c) Highly reflective material with rough limit (depth = 45 *μ*m).

**Figure 4:**
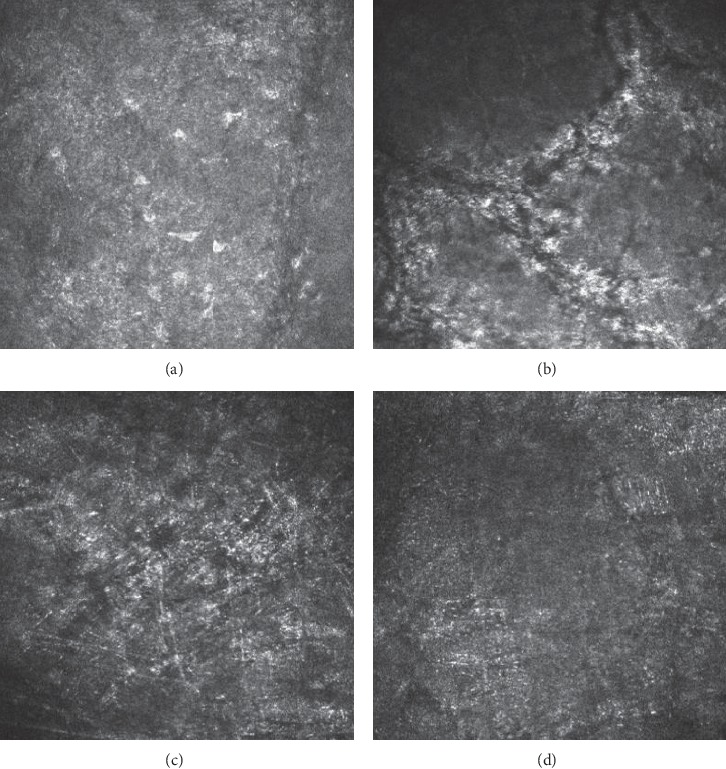
(a) Slightly high reflection (depth = 42 *μ*m). (b) High reflection with differently oriented dark striae (depth = 48 *μ*m). (c) Highly reflective microdots or needle-shaped material (depth = 373 *μ*m). (d) Cornea cells were not visible (depth = 530 *μ*m).

**Figure 5:**
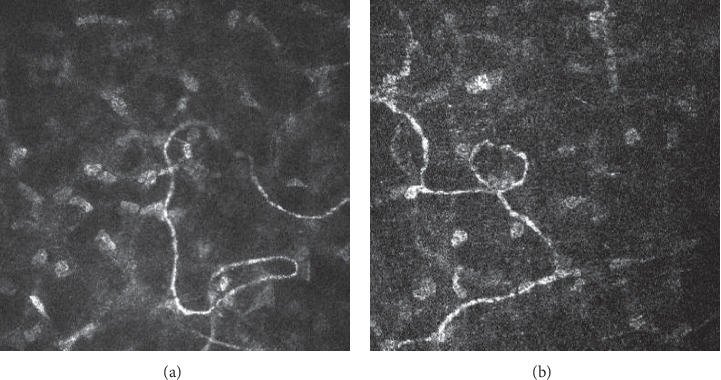
(a) Tortuous and thin stromal nerve (depth = 159 *μ*m). (b) Stromal nerve with bead-like changes (depth = 293 *μ*m).

In the epithelium of pterygium head, different number of inflammatory cells and dendritic cells were observed. Fibrous and blood vessel proliferation presented at the lower layer (Figure 2). Six patients (25%) had corneal degeneration around the head of pterygium, which was characterized by rough limit, infiltration of activated dendritic cells, and increased reflection rate of epithelial layer in IVCM images. (Figure 3). Of 24 eyes with pterygium, 20 (83.3%) had highly reflective microdots or needle-shaped material at the level of anterior to posterior stroma, 4 cases (16.7%) in the central cornea, 16 cases (66.7%) in the pterygium area, 13 cases (54.2%) in the region below pterygium, 17 cases (70.8%) in the region above pterygium, and 7 cases (29.2%) in the temporal side (Figure 4). It was difficult to observe stromal cells or endothelial cells in 6 eyes due to regional hyperreflectivity in the stromal layer. Stromal nerves were observed in 17 eyes with pterygium, of which 7 (42.2%) found thinning, 6 (35.3%) found tortuosity, and 1 (5.9%) found bead-like changes (Figure 5).

The density of corneal basal epithelial cells in the central region was 6497 ± 1776 cells/mm^2^, which was higher than that in the area adjacent to the head of pterygium (5580 ± 1294 cells/mm^2^, *P* < 0.001), the region above pterygium (6097 ± 1281 cells/mm^2^, *P*=0.049), and the lower region (5463 ± 1007 cells/mm^2^, *P*=0.001). The density of anterior stromal cells in the central region was 742 ± 243 cells/mm^2^, which was higher than that in the area near the head of pterygium (587 ± 189 cells/mm^2^, *P*=0.005), the region below pterygium (492 ± 159 cells/mm^2^, *P*=0.005), and the temporal cornea (574 ± 164 cells/mm^2^, *P*=0.003). The density of endothelial cells in the central cornea was 2398 ± 260 cells/mm^2^, which was higher than that in the area near the head of pterygium (2296 ± 231 cells/mm^2^, *P*=0.011) and the region below pterygium (2272 ± 400 cells/mm^2^, *P*=0.020). The density of dendritic cells in the central cornea was 53 ± 48 cells/mm^2^, which was lower than that in the area near the head of pterygium (250 ± 224 cells/mm^2^, *P*=0.001), the upper region (103 ± 47 cells/mm^2^, *P*=0.006), and the lower region (90 ± 48 cells/mm^2^, *P*=0.023). The CNFL in the center was higher than that in the pterygium head area, the upper region, and the lower region (Table 2). There was no significant difference in the number of cell parameters in the central cornea compared with the nasal cornea and the temporal cornea of the fellow eye (Table 3). Compared with the fellow eyes, the eyes with pterygium exhibited a significantly higher KM (*t* = 6.732, *P* < 0.001). The KM in eyes with pterygium was correlated with the percentage of corneal area invaded by pterygium. There was also a significant positive linear relationship between pterygium invasion and KM (*r* = 0.609, *P*=0.009).

## 5. Discussion

The pathogenesis of pterygium may be related to gene mutation, uncontrolled apoptosis, cytokines, viral infection, ultraviolet radiation, and other environmental factors [2]. In this study, the KM in eyes with pterygium was higher compared with fellow eyes and correlated with the percentage of corneal area invaded by pterygium, which is consistent with the existed study [3, 4]. In this study, only 1 case had pterygium invading the central corneal area. The density of basal epithelial cells in the central cornea of eyes with pterygium was significantly higher than that in the peripheries of pterygium, but there was no significant difference in cell density in the corresponding areas of fellow healthy eyes. Thus, the basal epithelial cell density of the eyes with pterygium was more affected than that of the fellow eyes. Pterygium is associated with an increase in tear osmolarity and abnormal tear film function [5]. Increased tear osmolarity may lead to a series of alterations in cell structure and inflammation, which may result in corneal epithelial surface damage [6, 7]. Due to mechanical stretching caused by pterygium, corneal epithelial cells can be twisted and elongated, and corneal basal cells at the edge of pterygium sometimes exhibit a columnar arrangement, which further reduces the density of epithelial cells [8]. Wang et al. [8, 9] found that the density of central corneal epithelial cells in eyes with pterygium was lower than that in the corresponding areas of fellow eyes. Papadia et al. [10] found that the density of epithelial cells was significantly lower in the pterygium group than in the control group. Moreover, the area of surface epithelial cells was significantly larger in the pterygium group than in the normal group, and the ratio of nucleus to cytoplasm was decreased.

Abnormal tear film may affect the nutritional and metabolic functions of the cornea, leading to changes in the density and morphology of corneal cells and nerves [11]. In this study, the density of anterior corneal stroma cells in the central cornea of eyes with pterygium was significantly higher than that in the peripheries of pterygium. Chui et al. [12, 13] found that pterygium can invade below the anterior elastic layer of the cornea, possibly affecting the morphology and quantity of corneal cells in the anterior stromal layer. Papadia et al. [10] reported a partial loss of central corneal stromal cells. Lacunae in the anterior stroma and an increase in the reflectivity of the extracellular matrix can also be observed in eyes with pterygium [9]. Wang et al. [8] found fewer stromal cells in the central cornea and cornea adjacent to pterygium in eyes with pterygium than in control eyes. However, there was no significant difference in the density of posterior stromal cells, which is consistent with this study. The highly reflective microdots and needle-shaped structures found in the stromal layers by IVCM had been observed in several different corneal inflammations. It is deduced to correspond to disorganized extracellular material, hyperreflective extracellular matrix, around degenerated collagen fiber bundles associated with deposition of lipid [14].

In this study, the ECD in the central cornea of eyes with pterygium was significantly higher than that in the peripheries of pterygium, but there was no significant difference in the cell density in the corresponding areas of fellow healthy eyes. Yang et al. [15] suggested that pterygium cells invading the anterior elastic layer could activate the expression of matrix metalloproteinases such as MMP-1, MMP-2, and MMP-9, thus affecting the connection of hemidesmosomes, which may lead to damage to corneal endothelial cells. In regard to corneal endothelial cells in pterygium, some studies noted [16–19] that the ECD was lower in the central or peripheral cornea of eyes with pterygium than in that of control eyes, whereas other studies [10] did not find any difference between eyes with pterygium and healthy eyes. Different results may be caused by different observation points. When pterygium invades the central cornea, the endothelial cells of the central cornea may be more significantly affected. We surmise that the central corneal area and the peripheral area of pterygium should be observed separately.

Dendritic cells are generally believed to play an important role in the initiation of immune response. Wang et al. [8] found that the density of dendritic cells was significantly higher in the pterygium group than in the control group, and the density in the pterygium body and head was significantly higher than that in the basal corneal epithelium. Labbé et al. [20] reported that the density of dendritic cells in the head and body of pterygium was closely related to the activity of pterygium. In our study, the density of dendritic cells in the peripheries of pterygium of eyes with pterygium was significantly higher than that in the central cornea, but there was no significant difference in the density of dendritic cells in the corresponding area of the healthy eyes. The results may indicate that the inflammatory reaction around the head of pterygium is more active than that in the other regions, but it could not be concluded that the differences are all caused by the horizontal position since the layer of dendritic cells is not recorded.

In this study, the length of central corneal subepithelial nerve fibers in pterygium patients was significantly longer than that in the pterygium area and the cornea surrounding pterygium. However, in the fellow eyes, the CNFL in central corneas was found longer than that in nasal corneas. Chances are that the CNFL in eyes with pterygium was affected not only by pterygium. Wang et al. [8] used IVCM to observe pterygium and found that the number and curvature of subepithelial nerve fibers increased in patients with primary monocular pterygium compared with those in fellow healthy eyes. Morphological changes, such as distortion, rupture, local protuberance, and formation of a circular structure were also observed, which was not consistent with this study. The changes in corneal subepithelial nerve fibers are affected not only by changes in tear film [11] but also by the secretion of interleukin-1 (IL-1) and IL-6 and ciliary neurotrophic factor (CNTF) stimulated by ultraviolet radiation [21, 22]. IL-1 is a key regulator of apoptosis and may be related to the apoptosis of prostromal cells. IL-1 can also induce the synthesis of nerve growth factor (NGF). Overexpression of NGF can promote nerve regeneration and increase the density of nerve fibers [23]. It is possible that the pterygium activity period was not evaluated in this study, and there was a difference between the nerve damage period and the regeneration period, which affected the results.

Overall, in this study, IVCM was used to observe the changes in cell density surrounding pterygium. This study is the first to evaluate the morphology and density of corneal cells on the temporal side of eyes with pterygium. However, this study had some limitations. For example, as a cross-sectional study, there is a lack of dynamic data on corneal cells, which needs further studies. IVCM could be used to observe histopathological changes during the development of pterygium, to study the characteristics of pterygium, and to improve the understanding of the pathogenesis of pterygium.

## Figures and Tables

**Table 1:** Basic demographic data of 24 patients.

	Eyes with pterygium	Fellow eyes	*P*
Mean corneal power (KM), (D)	44.684 ± 1.631	44.594 ± 1.645	0.433
Astigmatism (AST), (D)	2.486 ± 1.266	0.727 ± 0.401	<0.001
Percentage of pterygium on the cornea	12.05% ± 6.91%	—	—

**Table 2:** Differences in the number of cells and nerve parameters in the central cornea, area adjacent to the head of pterygium, region above pterygium, and region below pterygium.

Layers	Central cornea	Pterygium	Region above pterygium	Region below pterygium	Temporal cornea
Basal epithelial cells (cells/mm^2^)	6497 ± 1776	5580 ± 1294^*∗∗*^	6097 ± 1281^*∗*^	5463 ± 1007^*∗*^	6270 ± 1170
Anterior stromal cells (cells/mm^2^)	742 ± 243	587 ± 189^*∗∗*^	625 ± 154	492 ± 159^*∗∗*^	574 ± 164^*∗*^
Posterior stromal cells (cells/mm^2^)	435 ± 74	436 ± 99	403 ± 82	400 ± 83	390 ± 69
Endothelial cells (cells/mm^2^)	2398 ± 260	2296 ± 231^*∗*^	2276 ± 372	2272 ± 400^*∗*^	2300 ± 303
Dendritic cells (cells/mm^2^)	53 ± 48	250 ± 224^*∗∗*^	103 ± 47^*∗∗*^	90 ± 48^*∗*^	50 ± 41
CNFD (n/mm^2^)	12.14 ± 3.63	8.09 ± 3.63^*∗∗*^	9.36 ± 4.67^*∗*^	8.47 ± 4.88^*∗∗*^	9.95 ± 3.42^*∗*^
CNFL (mm/mm^2^)	30.76 ± 6.91	22.97 ± 8.10^*∗∗*^	26.04 ± 11.08	21.91 ± 9.38^*∗∗*^	27.02 ± 8.99
CNBD (n/mm^2^)	15.78 ± 15.50	8.40 ± 5.09	10.61 ± 5.78	14.42 ± 11.44	9.45 ± 9.01
CNFT	0.84 ± 0.86	1.11 ± 0.87	0.52 ± 0.59	0.96 ± 0.81	1.27 ± 1.08

^*∗*^ *P* < 0.05. ^*∗∗*^*P* < 0.01.

**Table 3:** Differences in the number of cell parameters in the central cornea, nasal cornea, nasal mid-peripheral cornea, and temporal cornea of the fellow eyes.

Layers	Central cornea	Nasal cornea	Superior nasal cornea	Below nasal cornea	Temporal cornea
Basal epithelial cells (cells/mm^2^)	6369 ± 801	6155 ± 975	6004 ± 727	5669 ± 489	6079 ± 562
Anterior stromal cells (cells/mm^2^)	649 ± 198	570 ± 186	575 ± 162	515 ± 107	594 ± 141
Posterior stromal cells (cells/mm^2^)	436 ± 115	430 ± 79	467 ± 83	409 ± 57	398 ± 92
Endothelial cells (cells/mm^2^)	2404 ± 216	2371 ± 212	2472 ± 247	2554 ± 303	2343 ± 250
Dendritic cells (cells/mm^2^)	35 ± 47	63 ± 65	64 ± 36	67 ± 66	49 ± 57
CNFD (n/mm^2^)	12.34 ± 4.58	9.40 ± 3.64	14.80 ± 6.76	11.25 ± 2.76	8.56 ± 3.85
CNFL (mm/mm^2^)	29.54 ± 13.14	21.55 ± 8.84^*∗∗*^	22.01 ± 9.61^*∗∗*^	33.69 ± 12.19	32.03 ± 4.69
CNBD (n/mm^2^)	12.37 ± 7.09	10.65 ± 8.85	14.06 ± 10.79	12.85 ± 15.94	9.53 ± 9.60
CNFT	1.09 ± 0.94	1.35 ± 1.23	0.79 ± 0.60	0.83 ± 0.53	1.19 ± 0.92

^*∗*^ *P* < 0.05. ^*∗∗*^*P* < 0.01.

## Data Availability

The data used to support the findings of this study are available from the corresponding author upon request.
